# Joint Association of Diet Index for Gut Microbiota and MVPA With Central Obesity: The Mediating Role of Biological Age

**DOI:** 10.1002/fsn3.71516

**Published:** 2026-02-02

**Authors:** Qi Zhou, Caifa Tang, Xin Pan, Zuyao Lu, Rujun Chen, Xiaohua Gong

**Affiliations:** ^1^ Department of Endocrinology and Metabolism The First Affiliated Hospital of Wenzhou Medical University Wenzhou Zhejiang China; ^2^ Wenzhou Medical University Wenzhou Zhejiang China; ^3^ Cixi Biomedical Research Institute Wenzhou Medical University Wenzhou Zhejiang China; ^4^ Department of Burn 906 Hospital of the Joint Logistics Team, PLA Wenzhou China; ^5^ National Key Clinical Specialty (Wound Healing) The First Affiliated Hospital of Wenzhou Medical University Wenzhou Zhejiang China

**Keywords:** biological age, central obesity, diet‐gut microbiota interaction, moderate‐to‐vigorous physical activity

## Abstract

This study examined the independent/joint effects of diet‐gut microbiota (DI‐GM) scores and moderate‐to‐vigorous physical activity (MVPA) on central obesity and mediation via biological aging. Using NHANES 2007–2018 data (17,012 adults), DI‐GM scores and MVPA (MET‐minutes/week) were assessed. Central obesity was defined as BMI ≥ 25 + waist‐height ratio ≥ 0.5. Biological age was measured via Klemera Doubal Method (KDM), phenotypic age (PA), and homeostasis disorder (HD). Multivariable logistic regression and mediation analyses evaluated associations. Each 1‐point DI‐GM increase reduced central obesity prevalence by 9% (OR = 0.91, 95% CI, 0.89%–0.94%, *p* < 0.001). Meeting MVPA recommendations (≥ 600 MET‐min/week) lowered prevalence by 18% (OR = 0.82, 0.71%–0.94%, *p* < 0.001). Participants with DI‐GM ≥ 6 + adequate MVPA had maximal risk reduction (OR = 0.60 vs. DI‐GM ≤ 4 + inadequate MVPA, 0.49%–0.75%, *p* < 0.001). Biological aging mediated 20.12% (KDM), 22.63% (PA), and 1.68% (HD) of DI‐GM's protective effects (*p* < 0.05), but not MVPA's effects. Stronger associations occurred in females, college‐educated individuals, and those with 7–8 h sleep (*p*‐interaction < 0.05). Higher DI‐GM scores and adequate MVPA significantly reduced central obesity prevalence, partially mediated by slower biological aging. Integrating gut microbiota‐targeted diets with physical activity may enhance obesity prevention.

## Graphical abstract

This study found that higher dietary index for gut microbiota (DI‐GM) scores and adequate moderate‐to‐vigorous physical activity (MVPA) were both independently associated with lower central obesity prevalence. Participants with both high DI‐GM (≥ 6) and recommended MVPA showed a 40% reduced risk of central obesity. Biological age partially mediated the association between DI‐GM and central obesity, highlighting the role of gut microbiota‐related dietary patterns in metabolic health.

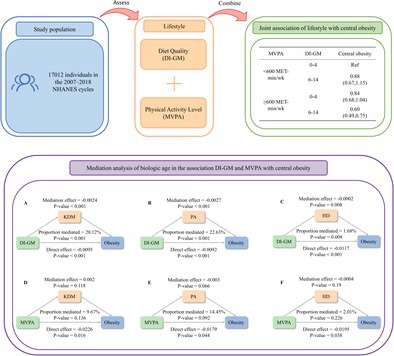

## 1 Introduction

In the United States, the prevalence of obesity has been consistently rising, with significant disparities across different socio‐economic and geographic groups. In the United States, 69% of adults are overweight or have obesity, and the global prevalence of obesity is increasing, with profound implications for healthcare systems and mortality rates (Wang et al. 2020; Malcolm et al. 2018). Obesity, especially central obesity, is closely associated with various adverse health outcomes. Studies have shown that central obesity has a significant link to the development of cardiovascular disease. Although obesity is linked to an increased incidence of cardiovascular disease, obese patients seem to have a better short‐term prognosis after acute coronary syndrome (ACS) (Kadakia et al. 2011). In addition, central obesity is closely associated with the development of metabolic syndrome (Olatunbosun et al. 2018). Central obesity is also associated with poor pregnancy outcomes. Studies have shown that pre‐pregnancy overweight/obesity and central obesity increase the risk of adverse pregnancy outcomes such as gestational diabetes mellitus, cesarean section, and macrosomia (Gao et al. 2017). This highlights the urgent need for effective, sustainable, and culturally tailored interventions to address the obesity epidemic.

While genetic predisposition contributes to obesity risk, modifiable lifestyle factors, particularly diet and physical activity, remain pivotal targets for prevention and intervention. The dietary index for gut microbiota (DI‐GM) identified 14 kinds of food or nutrients related to gut microbiota by reviewing 106 articles, with higher scores reflecting healthier gut microbiota (Kase et al. 2024). Additionally, DI‐GM positively correlated with urinary enterodiol and enterolactone (markers of gut microbiota diversity), indicating a link between this index and gut microbiota diversity. Therefore, the DI‐GM can effectively identify dietary patterns that are beneficial or harmful to gut microbiota. Increasing evidence has indicated that the gut microbiota is actively involved in the aging process (Ling et al. 2022). Cheng et al. revealed that by supplementation with the probiotic *Lactobacillus paracasei* PS23, the gut microbiota can be effectively adjusted, oxidative stress and inflammatory responses triggered by aging (Cheng et al. 2022).

Research shows that the composition and diversity of gut microbiota differ significantly between obese individuals and those with normal weight (Maruvada et al. 2017). Furthermore, the regulation of gut microbiota is considered a potential strategy for preventing and treating obesity. Probiotics, prebiotics, and synbiotics have shown some effectiveness in regulating gut microbiota and may help reduce weight by improving metabolic parameters (Vallianou et al. 2020). In addition, the impact of diet on gut microbiota is also a crucial aspect in obesity research.

Moderate‐to‐vigorous physical activity (MVPA) is widely recognized for its role in combating obesity and promoting overall health (Sabag et al. 2024). A study examining the influence of MVPA on adiposity and cardiorespiratory fitness in children found that vigorous physical activity is a significant predictor of reduced fatness and is significantly correlated with fitness levels (Parikh and Stratton 2011; Phillips et al. 2017). This suggests that engaging in higher intensity physical activities can lead to better health outcomes, potentially influencing biological age by slowing down the aging process at the cellular level.

Emerging evidence suggests that lifestyle factors such as dietary patterns and physical activity exert synergistic effects on metabolic health, yet their joint association of DI‐GM and MVPA with central obesity through biological aging mechanisms remains underexplored. Therefore, the objective of this study was to investigate the joint association of DI‐GM and MVPA with central obesity by analyzing adult data from the National Health and Nutrition Examination Survey (NHANES). Additionally, the study aimed to explore the potential mediating role of biological age in this association.

## 2 Methods

### 2.1 Population Under Investigation

**FIGURE 1:**
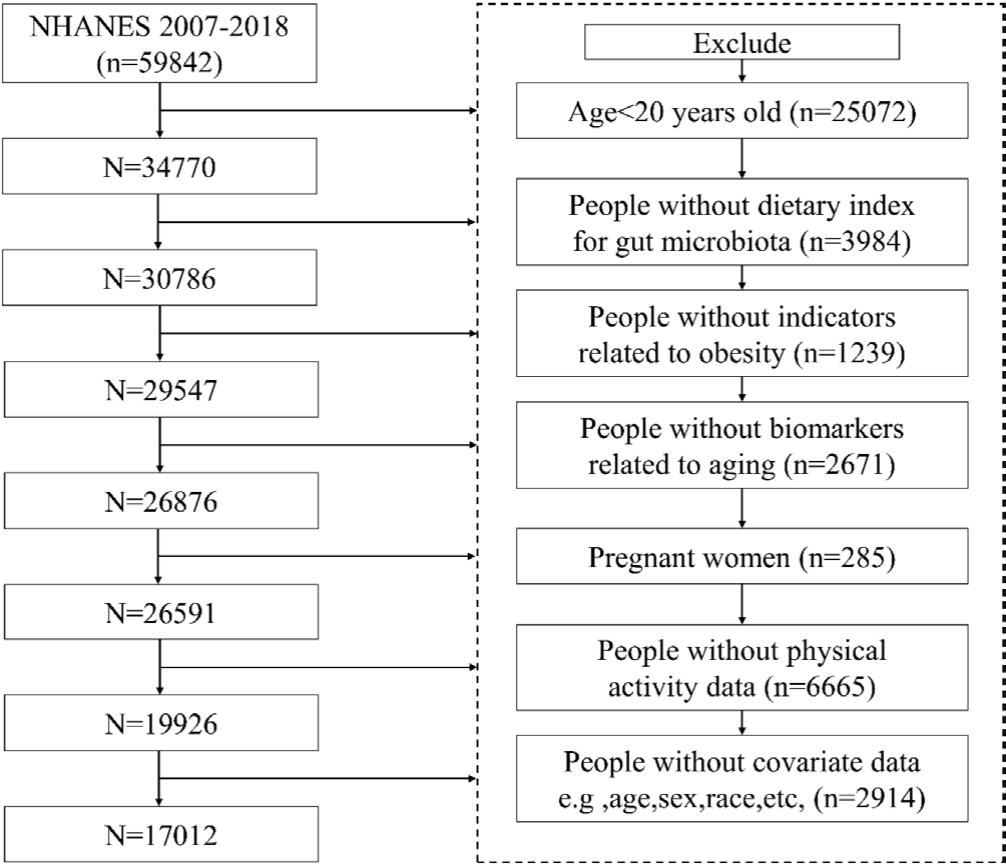
Study flow chart.

National Health and Nutrition Examination Survey (NHANES) is a continuous cross‐sectional study conducted by the National Center for Health Statistics (NCHS) using a complex, stratified, multistage probability sampling method to assess the health and nutritional status of the noninstitutionalized US national population. NHANES data are publicly accessible at https://wwwn.cdc.gov/nchs/nhanes/. NHANES protocols were approved by the Institutional Review Board of the NCHS, and written informed consent was obtained from all participants. Data from participants in the 2007–2018 NHANES cycles were analyzed, as these cycles provided information on DI‐GM, MVPA, biological age and obesity. In this study, participants aged 20 years or older were included, while participants under 20 years of age (*n* = 25,072), participants with incomplete data on DI‐GM (*n* = 3984), participants without indicators related to obesity (*n* = 1239), participants without biomarker data related to aging (*n* = 2671), pregnant women (*n* = 285), participants without MVPA data (*n* = 6665), and participants without complete covariates, such as age, sex, race, education, energy intake, smoking status, alcohol status, and disease history (*n* = 2914), were excluded. Finally, 17,012 participants were included in the study. Figure 1 details the inclusion and exclusion criteria.

### 2.2 The DI‐GM

The DI‐GM consists of 14 foods or nutrients, with beneficial components including fermented dairy, chickpeas, soybean, whole grains, fiber, cranberries, avocados, broccoli, coffee, and green tea, and unfavorable components including red meat, processed meat, refined grains, and high‐fat diet (≥ 40% energy from fat) (Kase et al. 2024). The DI‐GM score was calculated using 24‐h dietary recall data from NHANES 2007 to 2018. For beneficial components, a score of 1 was assigned if consumption was ≥ the sex‐specific median, otherwise 0 score; for unfavorable components, a score of 0 was assigned if consumption was ≥ the sex‐specific median or 40% (for high‐fat diet), otherwise 1 score. The DI‐GM score (ranges from 0 to 14) was the sum of these component scores and categorized into three groups: 0–4, 5, and ≥ 6 (Zhang et al. 2024; An et al. 2024).

### 2.3 Central Obesity

Compared with the general obesity represented by body mass index (BMI), abdominal obesity has been proposed to be a much stronger risk factor for CVD (Sanchez‐Lastra et al. 2024). Waist‐to‐height ratio (WHtR) is a better indicator of overfat and obesity when compared to waist circumference (WC) and waist hip ratio (Moltrer et al. 2022), and it has been recommended to measure obesity in the most recent United Kingdom obesity guidelines (Excellence NIfHaC 2025). Central obesity was defined as BMI ≥ 25 and WHtR ≥ 0.5.

### 2.4 Biological Age

At present, the most common indicators for evaluating biological age are the Klemera–Doubal Method (KDM), phenotypic age (PA), and homeostasis disorder (HD) (Zhu et al. 2024; Huang et al. 2024). In our study, we employed the “BioAge” R package for computing biological aging, which allowed us to implement various established aging models, including KDM, PA, and HD. The “BioAge” package is a comprehensive tool designed for the calculation of biological age based on quantifiable biomarkers. It integrates multiple algorithms that are widely recognized for their accuracy and reliability in aging research. The website https://github.com/dayoonkwon (accessed on September 30 2024) provides the relevant algorithms and R code form the “BioAge” package. The blood biochemical indexes were initially trained using data from NHANES III. In this study, 11 blood biomarkers were selected: alkaline phosphatase, total cholesterol, uric acid, albumin, creatinine, glycohemoglobin, white blood cell (WBC) count, lymphocyte percentage, mean cell volume, blood urea nitrogen, and red cell distribution width. The collection and processing of samples were conducted according to the detailed instructions outlined in the Laboratory/Medical Technologists Procedures Manual of the NHANES. Serum samples were stored at −30°C under freezing conditions before analysis. The measurement of biomarkers in the serum utilized two types of instruments: Beckman Synchron LX20 and Beckman Coulter UniCel D × C800 (Beckman Coulter, Brea, CA, USA), in accordance with specific biochemical methods. For example, alkaline phosphatase was measured through enzymatic rate methods, albumin via bichromatic digital endpoint methods, blood urea nitrogen through enzymatic conductivity rate methods, and cholesterol and creatinine utilizing timed‐endpoint and Jaffe rate methods, respectively. For uric acid, a timed endpoint method was utilized. The blood cell count was completed using the Beckman Coulter MAXM instrument at the Mobile Examination Centers (MECs). Glycated hemoglobin was measured using high‐performance liquid chromatography (HPLC). KDM used regression analysis to measure the specific biomarkers and chronological age of the reference population. PA was developed by analyzing various factors related to mortality risks to estimate death risk (Maruvada et al. 2017). The HD was used to determine the extent to which an individual's physiological measurement differed from the reference values derived from a young and healthy population. C‐reactive protein was excluded from the calculations due to its absence in NHANES 2011–2014. Previous studies have shown a high correlation between aging data computed with and without C‐reactive protein.

### 2.5 MVPA

Physical activity was quantified using metabolic equivalents (MET)–minutes, calculated as MET values multiplied by activity duration (minutes) following established protocols (Tian et al. 2022). MET minutes (defined as the amount of oxygen consumed while sitting at rest) were engaged in weekly and categorized into MVPA doses (MET‐min/wk). The MVPA dose was quantified in 2 categories: low (< 600 MET‐min/wk), moderate to high (≥ 600 MET‐min/wk) (Laird et al. 2023).

### 2.6 Covariates

Based on previous studies, the potential covariates included age, gender, race/ethnicity, education level, marital status, poverty income ratio (PIR), smoking status, alcohol drinking status, hypertension, diabetes mellitus (DM), and energy intake. Smoking status can be grouped into 3 categories: never (less than 100 cigarettes), former (more than 100 cigarettes but quit), and now (more than 100 cigarettes and currently smoke). Self‐reported drinking status was categorized as follows: never (consumed < 12 drinks in a lifetime), former (consumed ≥ 12 drinks in 1 year but not in the last year, or did not drink in the last year but consumed ≥ 12 drinks in a lifetime), now (consumed alcohol within the past 12 months). Sleep duration during the night was categorized as follows: < 7 and > 8 h per day, 7–8 h per day. Furthermore, the analysis considered the history of diseases such as cardiovascular disease (CVD), hypertension, and diabetes. Hypertension was diagnosed if systolic blood pressure (SBP) ≥ 140 mmHg or diastolic blood pressure (DBP) ≥ 90 mmHg was observed in three separate measurements. The diagnostic criteria for diabetes include doctors told you have diabetes, an HbA1c level greater than 6.5%, fasting blood glucose exceeding 7.0 mmol/L, random blood glucose more than 11.1 mmol/L, two‐hour OGTT blood glucose more than 11.1 mmol/L, use of diabetes medication or insulin.

### 2.7 Statistical Analysis

Following the guidance provided by the NHANES analysis manual, we analyzed the data of NHANES in six periods from 2007 to 2018 by using the sampling weight and also considering the main sampling units and stratification. In the descriptive analysis of baseline characteristics, continuous variables were expressed as weighted means and standard errors (SE), while categorical variables were conveyed through numerical counts and percentage frequencies (%). For continuous variables, we used the *t*‐test to assess the differences in baseline characteristics of participants with central obesity. The chi‐square test was employed to examine the differences among groups for categorical variables.

Independent associations of DI‐GM and MVPA as categorical variables with central obesity were examined by multivariable logistic regression analyses, adjusting for age, gender, education, marital status, race, PIR, smoking status, alcohol drinking, sleep duration, hypertension, DM, and energy intake when appropriate. Additionally, as continuous variables, the potential nonlinear relationship of DI‐GM and MET with central obesity was estimated by a restricted cubic spline (RCS) fitted in the fully adjusted logistic regression model.

Multivariable logistic regression models were employed to investigate the combined effects of DI‐GM and MVPA on the possibility of central obesity. First, we determined the interaction between DI‐GM and MVPA with central obesity by introducing interaction terms to evaluate whether the combined effect of DI‐GM and MVPA exceeds their cumulative effect on additive and multiplicative scales. For the additive scale interaction, we computed the relative excess risk due to interaction (RERI), the attributable proportion due to interaction (AP), and the synergy index (S). For the multiplicative interaction analysis, we compared models with versus without the DI‐GM × MVPA product term using likelihood ratio tests. Second, a joint analysis was conducted on four distinct groups delineated by categories of DI‐GM and MVPA, as follows: DI‐GM ≤ 4 and inadequate MVPA, DI‐GM ≤ 4 and recommended MVPA, DI‐GM ≥ 6 and inadequate MVPA, and DI‐GM ≥ 6 and recommended MVPA.

Subgroup analyses were analyzed based on age, sex, sleep duration, smoking status, alcohol drinking status, race/ethnicity, education level, marital status, PIR, hypertension, and DM. The analyses were replicated in the subset with complete data on all covariates. We additionally accounted for the use of antidepressants, antipsychotics, and corticosteroids in sensitivity analyses to assess the robustness of primary findings.

All data analyses were conducted using R version 4.2.1 (The R Foundation for Statistical Computing, Vienna, Austria). Statistical significance was defined as a two‐tailed *p*‐value less than 0.05.

## 3 Results

### 3.1 Study Population

Of the 59,842 participants, 17,012 were included in the final analyses (Figure 1). The baseline characteristics of the study population are presented in Table 1. Notably, individuals with central obesity were more likely to be older, male, have lower incomes and educational attainment, spend less time in recommended MVPA, have higher rates of current smoking and suboptimal sleep duration, have lower DI‐GM, have higher biologic age, and have higher WHtR and BMI (all *p* < 0.05).

**TABLE 1:** Participants characteristics.

Characteristics	Total (*n* = 17,012)	Normal (*n* = 5379)	Obesity (*n* = 11,633)	*p*
Age, mean (SD), years	45.74 (0.31)	42.34 (0.44)	47.40 (0.30)	< 0.0001
BMI, mean (SD), kg m^−2^	28.60 (0.10)	22.39 (0.04)	31.65 (0.10)	< 0.0001
WHtR, mean (SD)	0.58 (0.00)	0.49 (0.00)	0.63 (0.00)	< 0.0001
MET, mean (SD)	4914.24 (96.00)	5024.01 (131.25)	4860.43 (104.75)	0.21
KDM, mean (SD)	40.63 (0.28)	34.74 (0.35)	43.51 (0.30)	< 0.0001
PhenoAge, mean (SD)	42.88 (0.30)	38.12 (0.44)	45.21 (0.28)	< 0.0001
HD, mean (SD)	1.93 (0.01)	1.73 (0.01)	2.03 (0.01)	< 0.0001
DI‐GM score, mean (SD)	4.79 (0.03)	4.97 (0.04)	4.70 (0.03)	< 0.0001
Energy, mean (SD), kcal	2210.48 (9.92)	2237.38 (20.61)	2197.29 (11.63)	0.11
DI‐GM groups, *n* (%)				< 0.0001
0–4	44.19 (0.01)	40.05 (1.13)	46.22 (0.89)	
5	23.79 (0.01)	23.16 (0.84)	24.10 (0.58)	
6–14	32.02 (0.01)	36.79 (1.26)	29.69 (0.71)	
MVPA, *n* (%)				< 0.0001
< 600 MET‐min/wk	16.22 (0.01)	13.68 (0.75)	17.46 (0.51)	
≥ 600 MET‐min/wk	83.78 (0.02)	86.32 (0.75)	82.54 (0.51)	
Sex, *n* (%)				< 0.0001
Female	47.85 (0.01)	53.94 (0.89)	44.87 (0.59)	
Male	52.15 (0.01)	46.06 (0.89)	55.13 (0.59)	
Race, *n* (%)				< 0.0001
Mexican American	7.84 (0.01)	4.56 (0.43)	9.45 (0.88)	
Non‐Hispanic Black	9.38 (0.01)	8.17 (0.56)	9.98 (0.77)	
Non‐Hispanic White	70.25 (0.03)	72.36 (1.27)	69.22 (1.52)	
Other Hispanic	5.13 (0.00)	4.23 (0.44)	5.57 (0.42)	
Other Race	7.40 (0.00)	10.68 (0.73)	5.79 (0.34)	
Marital, *n* (%)				< 0.0001
Divorced	10.22 (0.00)	8.27 (0.46)	11.17 (0.47)	
Living with partner	8.33 (0.00)	9.10 (0.64)	7.95 (0.42)	
Married	54.76 (0.02)	49.12 (1.32)	57.53 (0.95)	
Never married	20.48 (0.01)	28.24 (1.28)	16.68 (0.78)	
Separated	2.12 (0.00)	1.85 (0.22)	2.26 (0.18)	
Widowed	4.09 (0.00)	3.43 (0.32)	4.41 (0.27)	
PIR, *n* (%)				0.15
Low	13.35 (0.01)	14.21 (0.98)	12.92 (0.65)	
Moderate	33.32 (0.01)	31.86 (1.15)	34.04 (0.94)	
High	53.33 (0.02)	53.93 (1.43)	53.03 (1.25)	
Education level, *n* (%)				< 0.0001
Less than 12th grade	11.84 (0.01)	10.34 (0.75)	12.57 (0.61)	
High school diploma	22.19 (0.01)	19.35 (0.92)	23.58 (0.77)	
Some college education	33.00 (0.01)	30.94 (1.09)	34.02 (0.68)	
College graduate and above	32.97 (0.02)	39.37 (1.45)	29.84 (1.09)	
Alcohol, *n* (%)				< 0.0001
Never	9.06 (0.01)	8.62 (0.72)	9.28 (0.60)	
Former	10.63 (0.01)	8.19 (0.59)	11.83 (0.47)	
Now	80.31 (0.02)	83.19 (0.96)	78.89 (0.87)	
Smoke, *n* (%)				< 0.0001
Never	55.65 (0.02)	57.59 (1.26)	54.70 (0.79)	
Former	24.87 (0.01)	19.30 (0.88)	27.60 (0.76)	
Now	19.48 (0.01)	23.12 (1.14)	17.70 (0.54)	
Sleep duration, *n* (%)				< 0.001
< 7 and > 8 h per day	44.53 (0.01)	41.66 (1.20)	45.93 (0.68)	
7–8 h per day	55.47 (0.02)	58.34 (1.20)	54.07 (0.68)	
Hypertension, *n* (%)				< 0.0001
No	65.96 (0.02)	80.46 (0.74)	58.86 (0.78)	
Yes	34.04 (0.01)	19.54 (0.74)	41.14 (0.78)	
Diabetes, *n* (%)				< 0.0001
No	92.07 (0.03)	97.17 (0.31)	89.57 (0.42)	
Yes	7.93 (0.00)	2.83 (0.31)	10.43 (0.42)	
CVD, *n* (%)				< 0.0001
No	93.46 (0.03)	95.56 (0.37)	92.43 (0.37)	
Yes	6.54 (0.00)	4.44 (0.37)	7.57 (0.37)	

*Note:* Continuous variables are presented as weighted mean (SD), whereas categorical variables are presented as actual frequency (weighted percentage [%]). Differences in Continuous variables and categorical variables distributed baseline characteristics were compared using the *t*‐test and the chi‐square, respectively. The DI‐GM categorized into three groups: 0–4, 5, and 6–14. “*n*” represents unweighted counts from the actual sample size.

Abbreviations: BMI, body mass index; CVD, cardiovascular disease; DI‐GM, dietary index for gut microbiota; HD, homeostasis disorder; KDM, Klemera–Doubal Method; MET, metabolic equivalents multiplied by minutes; MVPA, moderate‐to‐vigorous physical activity; PIR, poverty income ratio; SD, standard deviation; WHtR, waist to height ratio. Central obesity was defined as BMI ≥ 25 and WHtR ≥ 0.5.

### 3.2 Independent Associations of DI‐GM and MVPA With Central Obesity

As presented in Table 2, each 1‐point increment in the DI‐GM score was associated with a 10% reduction in central obesity prevalence in the crude model (odds ratio [OR]: 0.90; 95% confidence interval [CI], 0.88–0.93; *p* < 0.001). After full adjustment for covariates, this association remained significant with a 9% risk reduction (OR: 0.91; 95% CI, 0.89–0.94; *p* < 0.001). When categorizing DI‐GM scores, participants in the DI‐GM ≥ 6 group exhibited a 27% lower risk of central obesity compared to lower‐scoring groups in the fully adjusted model (OR: 0.73; 95% CI, 0.64–0.83; *p* < 0.001) (Table 2). When categorizing DI‐GM scores, participants in the DI‐GM ≥ 6 group exhibited a 27% lower risk of central obesity compared to lower‐scoring groups in the fully adjusted model (OR: 0.82; 95% CI, 0.71–0.94; *p* = 0.01) (Table 2).

**TABLE 2:** Independent associations of DI‐GM and physical activity with central obesity.

Characteristics	Case/*N*	Crude model		Model 1		Model 2	
		OR (95% CI)	*p*	OR (95% CI)	*p*	OR (95% CI)	*p*
DI‐GM score	11,633/17012	0.90 (0.88,0.93)	< 0.0001	0.90 (0.88,0.93)	< 0.0001	0.91 (0.89,0.94)	< 0.0001
DI‐GM group							
0–4	5638/8001	1 (reference)		1 (reference)		1 (reference)	
5	2721/3949	0.90 (0.80,1.02)	0.10	0.92 (0.80,1.04)	0.18	0.94 (0.83,1.08)	0.38
6–14	3274/5062	0.70 (0.62,0.79)	< 0.0001	0.70 (0.62,0.79)	< 0.0001	0.73 (0.64,0.83)	< 0.0001
*p* for trend			< 0.0001		< 0.0001		< 0.0001
MVPA							
< 600 MET‐min/wk	2154/2993	1 (reference)		1 (reference)		1 (reference)	
≥ 600 MET‐min/wk	9479/14019	0.75 (0.65,0.86)	< 0.0001	0.78 (0.67,0.90)	0.001	0.82 (0.71,0.94)	0.01

*Note:* Data are presented as odds ratios (95% confidence intervals). The crude model was not adjusted for any covariates. Model 1 = age, sex, race/ethnicity, marital, PIR, education level, alcohol, smoke, sleep duration. Model 2 = model 1 + (Hypertension, diabetes, CVD). The DI‐GM score categorized into DI‐GM groups: 0–4, 5, and 6–14.

Abbreviations: CI, confidence Interval; DI‐GM, dietary index for gut microbiota; MVPA, moderate‐to‐vigorous physical activity; OR, odds ratios.

**FIGURE 2:**
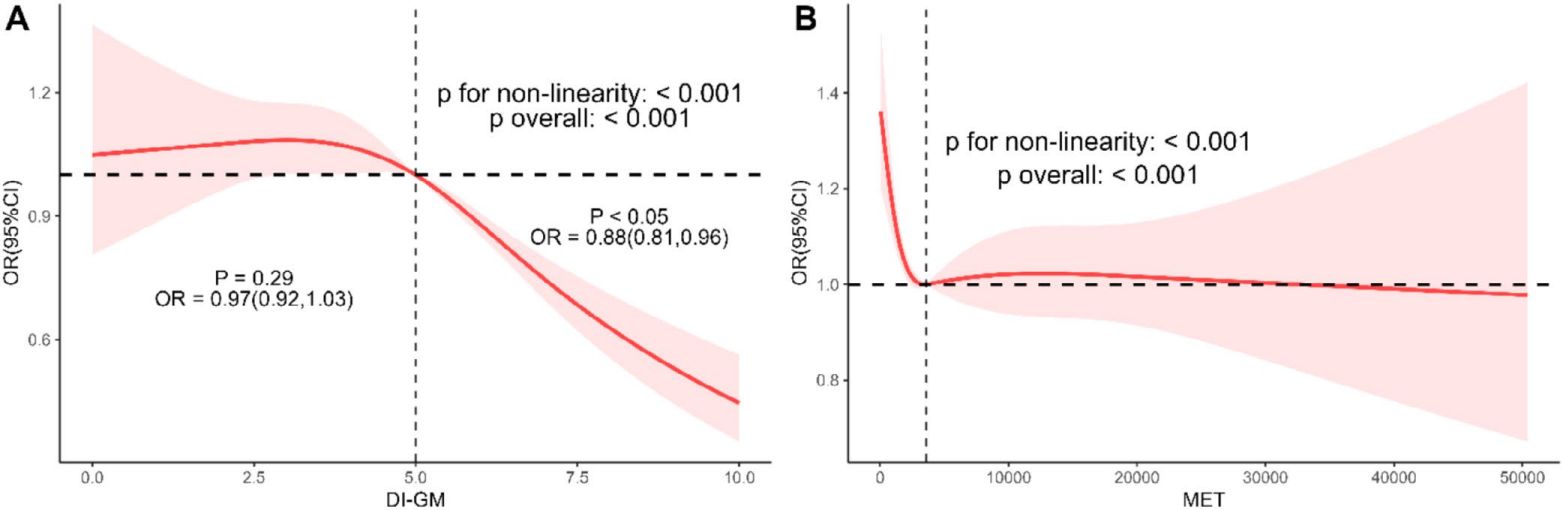
Independent associations of DI‐GM and MVPA with central obesity among the NHANES 2007–2018 participants by RCS. (A) Nonlinear association between DI‐GM score and central obesity. (B) Nonlinear association between MET and central obesity. The model was adjusted for age, gender, education, marital status, race, PIR, smoking status, alcohol drinking, sleep duration, hypertension, DM, CVD, and energy intake.

Nonlinear associations were further investigated using restricted cubic splines (RCS) with 4 knots. After multivariable adjustment, both DI‐GM score and physical activity demonstrated significant nonlinear relationships with central obesity prevalence (*P* for all < 0.001, non‐linear *p* < 0.001 for both) (Figure 2). Specifically, a reverse L‐shaped association was observed between DI‐GM score and central obesity, with prevalence decreasing sharply when DI‐GM exceeded the inflection point at 5.00 (Figure 2A). Conversely, MVPA exhibited an L‐shaped dose–response pattern, showing maximal protective effects below the threshold of 9656.50 MET‐min/week.

### 3.3 Joint Association of DI‐GM and MVPA With Central Obesity

Joint effect analyses revealed that participants with both DI‐GM ≤ 4 and inadequate MVPA exhibited the highest prevalence of central obesity (reference group). Compared to this reference group, those achieving DI‐GM ≥ 6 combined with recommended MVPA showed a 40% risk reduction (OR: 0.60; 95% CI, 0.49–0.75, *p* < 0.001) (Table 3).

**TABLE 3:** Joint association of DI‐GM and physical activity with central obesity among participants.

Characteristics			Crude model		Model 1		Model 2	
MVPA	DI‐GM	Case/*N*	OR (95% CI)	*p*	OR (95% CI)	*p*	OR (95% CI)	*p*
< 600 MET‐min/wk	0–4	1035/1400	1 (reference)		1 (reference)		1 (reference)	
	6–14	617/894	0.85 (0.65,1.11)	0.23	0.87 (0.66,1.14)	0.31	0.88 (0.67,1.15)	0.34
≥ 600 MET‐min/wk	0–4	4603/6601	0.78 (0.64,0.95)	0.02	0.83 (0.67,1.02)	0.08	0.84 (0.68,1.04)	0.11
	6–14	2657/4168	0.53 (0.43,0.65)	< 0.0001	0.57 (0.46,0.70)	< 0.0001	0.60 (0.49,0.75)	< 0.0001
*p* for trend				0.88		0.974		0.993

*Note:* Data are presented as odds ratios (95% confidence intervals). The crude model was not adjusted for any covariates. Model 1 = age, sex, race/ethnicity, marital, PIR, education level, alcohol, smoke, sleep duration. Model 2 = model 1 + (Hypertension, diabetes, CVD). Participants were categorized into four groups based on combined DI‐GM and MVPA with MVPA: No and DI‐GM: 0–4 designated as the reference.

Additive and multiplicative interaction analyses between DI‐GM and MVPA demonstrated no statistically significant effect modification on obesity prevalence (relative excess risk due to interaction [RERI] = −0.19, 95% CI, −0.71 to 0.28; multiplicative interaction *p* > 0.05, Table 4).

**TABLE 4:** Interaction effect analysis of DI‐GM and MVPA in relation to central obesity.

Additive interactive				Multiplicative interactive	*p*
Measure	Estimate	Lower	Upper	OR (95% CI)	
RERI	−0.19	−0.71	0.28	0.82 [0.59, 1.13]	> 0.05
AP	−0.11	−0.47	0.14		
S	0.78	0.42	1.43		

*Note:* The model was adjusted for age, gender, education, marital status, race, PIR, smoking status, alcohol drinking, sleep duration, hypertension, DM, CVD, and energy intake.

Abbreviations: AP, the attributable proportion due to interaction; RERI, the relative excess risk due to interaction; S, the synergy index.

### 3.4 Mediation and Subgroup Analyses

**FIGURE 3:**
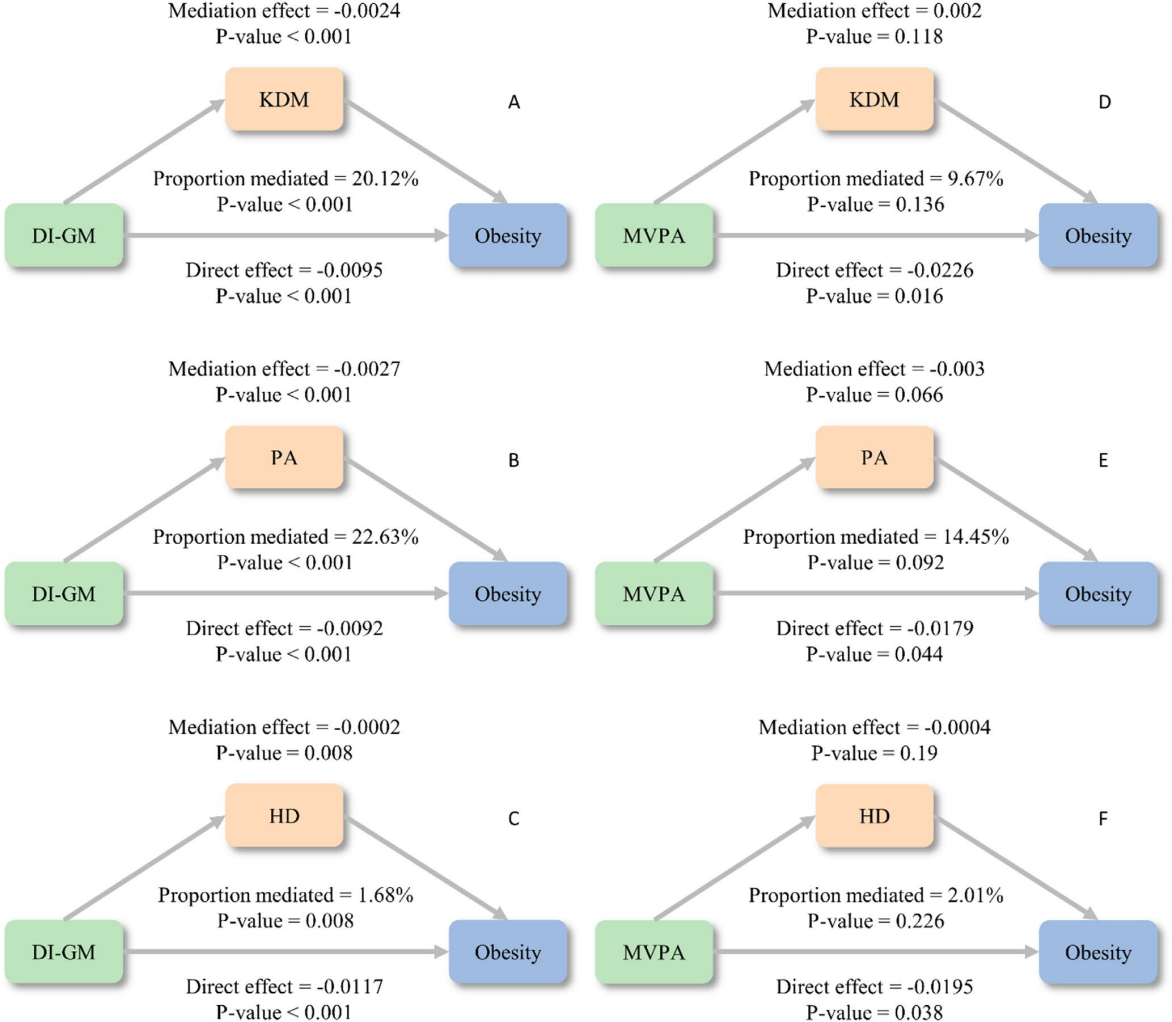
Mediation analysis of biologic age in the association between DI‐GM and MVPA with central obesity. (A–C) The mediation effect of biologic age in the association between DI‐GM and obesity. (D–F) The mediation effect of biologic age in the association between MVPA and obesity. The model was adjusted for age, gender, education, marital status, race, PIR, smoking status, alcohol drinking, sleep duration, hypertension, DM, CVD, and energy intake. DI‐GM, dietary index for gut microbiota; HD, homeostasis disorder; KDM, Klemera–Doubal method; MVPA, moderate‐to‐vigorous physical activity; PA, PhenoAge.

**FIGURE 4:**
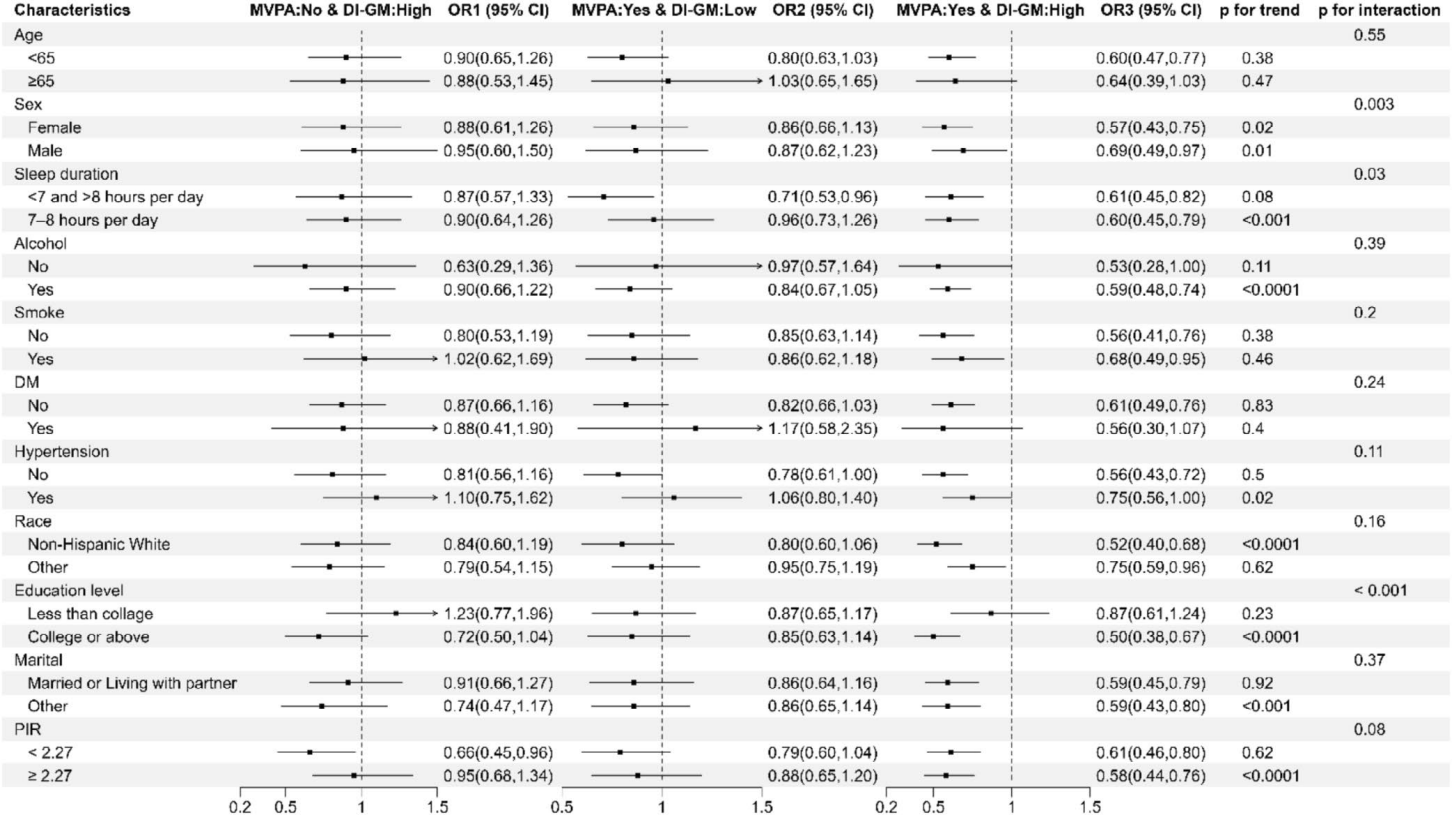
Subgroup analysis of the association between combined effects of DI‐GM and MVPA with central obesity. All models were adjusted for age, gender, education, marital status, race, PIR, smoking status, alcohol drinking, sleep duration, hypertension, DM, CVD, and energy intake. If the factor was subgroup stratified, it was not adjusted.

Mediation analyses revealed significant indirect effects of biological aging in the DI‐GM and central obesity association. KDM mediated 20.12% (*p* < 0.001), PA 22.63% (*p* < 0.001), and HD 1.68% (*p* = 0.008) of the total effect (Figure 3A–C). No significant mediation was observed for MVPA effects through biological aging mechanisms (*p* > 0.05; Figure 3D–F). Subgroup analyses were conducted by age, sex, sleep duration, smoking status, alcohol drinking status, race/ethnicity, education level, marital status, PIR, and diagnosis of hypertension and diabetes. Notably, gender, sleep duration, and education level had an interaction with DI‐GM ≥ 6 and recommended MVPA in relation to central obesity. DI‐GM ≥ 6 and recommended MVPA significantly associated with reduced central obesity prevalence in participants females, those with sleep duration with 7–8 h/day group, and education level with college or above (all *p* for interaction < 0.05, Figure 4). However, there was no significant interaction between DI‐GM ≥ 6 and recommended MVPA with other factors (all *p* for interaction > 0.05, Figure 4).

### 3.5 Sensitivity Analyses

In the fully adjusted model, the results remained consistent with the primary findings after including each medication individually and after simultaneously adjusting for the use of all medications. Compared with the reference group, participants with a DI‐GM score ≥ 6 combined with recommended levels of MVPA still exhibited a 40% lower risk (Table 5).

**TABLE 5:** Sensitivity analyses.

Characteristics		Model 2+ antidepressant		Model 2+ antipsychotics		Model 2+ corticosteroids		Model 2+ all medications	
MVPA	DI‐GM	OR (95% CI)	*p*	OR (95% CI)	*p*	OR (95% CI)	*p*	OR (95% CI)	*p*
< 600 MET‐min/wk	0–4	1 (ref)	1 (ref)	1 (ref)	1 (ref)	1 (ref)	1 (ref)	1 (ref)	1 (ref)
	6–14	0.87 (0.66, 1.15)	0.33	0.87 (0.66, 1.15)	0.32	0.88 (0.66, 1.15)	0.34	0.88 (0.67, 1.16)	0.36
≥ 600 MET‐min/wk	0–4	0.84 (0.68, 1.05)	0.12	0.84 (0.68, 1.05)	0.12	0.84 (0.68, 1.05)	0.12	0.85 (0.68, 1.05)	0.13
	6–14	0.60 (0.48, 0.75)	< 0.0001	0.60 (0.48, 0.74)	< 0.0001	0.60 (0.48, 0.74)	< 0.0001	0.60 (0.48, 0.75)	< 0.0001
*p* for trend			0.906		0.904		0.921		0.904

*Note:* Data are presented as odds ratios (95% confidence intervals). Model2 is the fully adjusted model.

## 4 Discussion

Obesity, particularly central obesity, has become a major public health issue worldwide. In the United States, obesity prevalence in the United States remains stubbornly persistent, so more effective strategies and measures are still needed to address the issue of obesity (Elmaleh‐Sachs et al. 2023). Dietary modification plays an important role in obesity treatment. Weight loss diets are available that include various permutations of energy restriction, macronutrients, foods, and dietary intake patterns (Ariana et al. 2021). The association between DI‐GM and central obesity was explored in depth using the NHANES database. The logistic regression model revealed that the DI‐GM score was negatively correlated with central obesity. DI‐GM is a novel dietary index to assess the quality of diets relevant to maintaining a healthy gut microbiota. Recently, many studies explored the association of DI‐GM with depression (Zhang et al. 2024), diabetes (Wu et al. 2025), non‐alcoholic fatty liver disease (Zheng et al. 2025), stroke (Liu and Huang 2025), and chronic kidney disease (Xiao et al. 2025). Chu et al. found that BMI showed a significant downward trend with the increase of DI‐GM score after adjusting for all covariates. Compared with the lowest DI‐GM score, the β value of DI‐GM and BMI in highest levels was −0.60 (−1.00, −0.20) (An et al. 2024). In our study, our results revealed that among adults aged ≥ 20 years, DI‐GM score was significantly and negatively associated with central obesity prevalence across all models, with each 1‐point increase corresponding to 10% and 9% reductions, respectively. The logistic regression model revealed that compared to the participants with DI‐GM ≤ 4, the prevalence of central obesity in those with DI‐GM ≥ 6 was significantly reduced. The RCS analysis further showed the dose–response relationship between DI‐GM score and central obesity, indicating that the association between DI‐GM and central obesity was nonlinear.

Physical activity is vital for weight loss (Tongyu et al. 2023; Hui et al. 2025). Our study showed similar results. We found that recommended MVPA reduced 18% central obesity prevalence compared to those without recommended MVPA after the fully adjustments. To our knowledge, there is no study investigating the joint effects of DI‐GM and MVPA on central obesity. In our study, in fully adjusted models, adherence to both DI‐GM ≥ 6 and recommended MVPA was associated with a 40% lower prevalence of central obesity compared to the combined exposure of DI‐GM ≤ 4 and inadequate MVPA. Furthermore, our study showed there were no significant interactions between the effects of DI‐GM and MVPA on central obesity. Stamatakis et al. demonstrated that combined improvements in physical activity and diet quality were associated with a 35% lower risk of obesity incidence (adjusted OR = 0.65, 95% CI, 0.48–0.89) (Ahmadi et al. 2023).

Interaction and subgroup analysis played a crucial role in deeply exploring the association between various factors. The joint association of DI‐GM and MVPA with central obesity may also be related to gender, sleep status, and education level. Among participants with normal sleep status, the combined effects of higher DI‐GM and recommended MVPA were associated with a greater reduction in the prevalence of central obesity. This finding might be because the abnormal sleep duration leads to the risk of obesity. Growing evidence from both epidemiological and laboratory‐based studies suggests sleep curtailment is a new risk factor for the development of obesity (Kelsie et al. 2024; Onge 2017).

To further investigate the potential mechanisms underlying DI‐GM, MVPA, and central obesity, we conducted a mediation analysis using biologic age as an intermediary factor. The relationship between aging and obesity is a complex and multifaceted issue. In recent years, more and more studies have revealed how aging promotes the occurrence of obesity (Shuji et al. 2025; Daniela et al. 2017). Biologic age reflects the cumulative effects of multisystemic aging (Jianhua et al. 2024). Our analysis demonstrated that biological age significantly mediated the association between DI‐GM and central obesity; however, no mediating effect of biological age was observed in the relationship between MVPA and central obesity. Chu et al. demonstrated that a higher DI‐GM score is associated with a lower risk of accelerated aging (An et al. 2024). Galkin and his colleagues found that keystone members of the gut community, such as Bacteroides, Eubacterium, and Bifidobacterium, have the greatest effect on age prediction and can be considered as potential aging biomarkers (Galkin et al. 2020). In addition, an animal study confirmed that *Akkermansia muciniphila* is sufficient to exert beneficial effects to enhance healthspan and lifespan in mouse models, possibly due to its beneficial effect of reestablishing a healthy gut microbiota (Bárcena et al. 2019). Correction of accelerated aging‐associated gut dysbiosis suggested a link between aging and the gut microbiota. The gut microbiota may accelerate aging through mechanisms such as immunosenescence (immune system decline) (Fulop et al. 2017) and inflammaging (low‐grade chronic inflammation) (Franceschi et al. 2018).

The differences in mediation proportions observed among the three biological age measures (KDM: 20.1%, PhenoAge: 22.6%, HD: 1.7%) likely reflect fundamental distinctions in their underlying construction and biological sensitivity. KDM and PhenoAge are composite indices derived from multiple metabolic, inflammatory, and hematological biomarkers closely related to obesity, diet, and physical activity (Kwon and Belsky 2021). Consequently, these measures are more responsive to lifestyle‐related metabolic changes. In contrast, HD primarily quantifies deviations from physiological homeostasis relative to a young and healthy reference population (Li et al. 2015). It focuses more on systemic dysregulation than on metabolic aging, which may explain its weaker mediation effect in the link between DI‐GM, MVPA, and central obesity.

We additionally performed sensitivity analyses accounting for the use of antidepressants, antipsychotics, and corticosteroids to evaluate the robustness of our main findings. These medications are known to promote weight gain and affect appetite regulation. After including these medications, the associations remained stable, confirming the robustness of our findings, indicating that our main finding were not driven by these pharmacological confounders. However, several medical conditions known to cause weight gain and metabolic alterations, such as hypothyroidism, sleep apnea, and polycystic ovary syndrome (PCOS), could not be adequately adjusted for due to insufficient or missing data in NHANES. These conditions may contribute to residual confounding, as individuals with such disorders often have altered energy metabolism, reduced physical activity, and poorer dietary quality (Chaker et al. 2017; Cena et al. 2020). In addition, confounders leading to pathological weight loss and cachexia were not explicitly accounted for, including hyperthyroidism, active malignancy, chronic infectious diseases (e.g., tuberculosis), and advanced chronic organ failure (heart, lung, liver, or kidney failure). Individuals with these conditions are less likely to be classified as having central obesity but often present with poor appetite, low diet quality (potentially low DI‐GM), and markedly reduced physical activity (low MVPA) due to disease burden. Their inclusion in the non–central obesity reference group may bias the observed associations toward the null, potentially leading to an underestimation of the protective effects of higher DI‐GM and adequate MVPA. Taken together, the coexistence of conditions causing weight gain and those causing pathological weight loss highlights a two‐sided residual confounding issue, which should be considered when interpreting the magnitude of the observed associations.

Another limitation is the lack of geographical and climatic data in NHANES. The United States spans diverse climates and cultures that influence gut microbiota, dietary patterns, and physical activity. Recent evidence suggests that temperature and humidity can shape gut microbial composition and appetite regulation. Because NHANES does not include detailed geographic identifiers, residual confounding from regional or environmental factors cannot be fully excluded (Wu et al. 2024). Therefore, our results should be interpreted with caution, acknowledging that unmeasured confounders might partially influence the observed associations.

Nonetheless, the present study has several limitations. First, the cross‐sectional design cannot establish causality. Longitudinal studies and randomized controlled trials are needed to confirm causal associations. Secondly, the DI‐GM was developed based on literature‐derived dietary components rather than directly measured gut microbiota profiles, which may not fully capture individual variations in microbiome composition or function (Kase et al. 2024). Moreover, both DI‐GM and MVPA are derived from self‐reported measures, making them susceptible to recall and social desirability bias. Thirdly, as in many studies, the possibility of confounding effects due to measurement error residuals from unmeasured variables or unknown confounders cannot be completely ruled out.

## 5 Conclusion

Our findings demonstrate that both DI‐GM and MVPA exhibit significant associations with central obesity prevalence. Notably, the synergistic combination of DI‐GM ≥ 6 and recommended MVPA levels showed a more pronounced protective effect against central obesity, particularly among females and individuals maintaining normal sleep patterns. Biological age partially mediated the DI‐GM and central obesity association, whereas no significant mediation effect was observed for MVPA.

## Author Contributions

**Qi Zhou:** conceptualization (equal), investigation (equal), methodology (equal). **Caifa Tang:** data curation (equal), formal analysis (equal), writing – original draft (equal), writing – review and editing (equal). **Xin Pan:** resources (equal), writing – review and editing (equal). **Zuyao Lu:** resources (equal), writing – review and editing (equal). **Rujun Chen:** conceptualization (equal), methodology (equal), resources (equal), supervision (equal). **Xiaohua Gong:** conceptualization (equal), methodology (equal), resources (equal), supervision (equal), validation (equal), visualization (equal), writing – review and editing (equal).

## Funding

This study was supported by the Medical Science and Technology Project of Zhejiang Province (grant number: 2024KY1249) and the Science and Technology Project of Wenzhou (grant No. Y20240216).

## Conflicts of Interest

The authors declare no conflicts of interest.

## Data Availability

The data that support the findings of this study are openly available in National Health and Nutrition Examination Survey (NHANES) at https://www.cdc.gov/nchs/nhanes/.
